# Clinicopathological significance of MYL9 expression in pancreatic ductal adenocarcinoma

**DOI:** 10.1002/cnr2.1582

**Published:** 2021-11-24

**Authors:** Katsunori Matsushita, Shogo Kobayashi, Hirofumi Akita, Masamitsu Konno, Ayumu Asai, Takehiro Noda, Yoshifumi Iwagami, Tadafumi Asaoka, Kunihito Gotoh, Masaki Mori, Yuichiro Doki, Hidetoshi Eguchi, Hideshi Ishii

**Affiliations:** ^1^ Department of Gastroenterological Surgery Graduate School of Medicine, Osaka University Suita Osaka Japan; ^2^ Center of Medical Innovation and Translational Research Graduate School of Medicine, Osaka University Suita Osaka Japan; ^3^ Artificial Intelligence Research Center The Institute of Scientific and Industrial Research, Osaka University Ibaraki Osaka Japan; ^4^ Institute of Scientific and Industrial Research Osaka University Ibaraki Osaka Japan; ^5^ Department of Surgery and Science Graduate School of Medical Sciences, Kyushu University Fukuoka Japan

**Keywords:** apoptosis, cell proliferation, myosin light chains, pancreatic ductal adeno carcinoma

## Abstract

**Background:**

Pancreatic ductal adenocarcinoma is one of the most aggressive malignancies, and often involves invasion and distant metastasis from the early tumor stages. Myosin II reportedly plays a key role in regulating tumor progression and metastasis.

**Aims:**

We examined whether myosin regulatory light polypeptide 9 (MYL9) regulates cancer cell proliferation.

**Methods and results:**

To investigate the expression pattern and clinical significance of MYL9 in pancreatic ductal adenocarcinoma, we performed immunohistochemical analysis of samples collected from 101 patients with pancreatic ductal adenocarcinoma. The expression of MYL9 was investigated to evaluate its functional role and contribution to proliferation and apoptosis in pancreatic ductal adenocarcinoma cells in vitro. The results showed that MYL9 was predominantly expressed in the cytoplasm and membrane of pancreatic ductal adenocarcinoma cells. Multivariate analysis indicated that MYL9 acted as an independent prognostic factor for overall survival and distant metastasis‐free survival. MYL9 expression was strongly associated with malignancy in in vitro analyses, including proliferation and anti‐apoptotic activities.

**Conclusions:**

Our findings suggest that MYL9 is an independent prognostic factor of pancreatic ductal adenocarcinoma. MYL9 is a crucial biomarker and potential therapeutic target for pancreatic ductal adenocarcinoma.

## 1 INTRODUCTION

Pancreatic ductal adenocarcinoma (PDAC) is a fatal neoplasm with a 5‐year survival rate of <5%.^1^, ^2^, ^3^ It is predicted that PDAC will be the second major cause of tumor‐associated mortalities in the United States by 2030.^4^ Surgical resection is currently the only curative treatment, and due to rapid tumor growth and metastasis, only 20% of patients are eligible for resection.^5^ In the light of these challenges, surgeons have developed novel measures to manage PDAC.^6^

Myosins convert the energy released after ATP hydrolysis into force and play important roles in the regulation of tumor progression and metastasis.^7^ Members of the myosin superfamily have been shown to be associated with tumor progression.^8^, ^9^ Myosin II was the first myosin to be studied biochemically.^7^ It is composed of six polypeptide chains: two heavy chains and two sets of paired light chains, including the essential and regulatory light chains.^10^ Myosin regulatory light polypeptide 9 (MYL9) has been shown to be important for cytoskeleton dynamics, experimental metastasis, and invasion.^11^ Although a large amount of evidence regarding the role of MYL9 in the promotion of tumor invasion and metastasis has been uncovered, the clinical significance of MYL9 in human tissues differs depending on the tumor type.^12^, ^13^ Total expression of MYL9 has been shown to be downregulated in tumor tissues, compared with normal tissues, in colon cancer, noninvasive small‐cell lung cancer, and bladder cancer.^14^, ^15^, ^16^ The low expression of MYL9 in stromal cells was associated with malignant progression and recurrence‐free survival in patients with prostate cancer.^17^ Low MYL9 expression has been associated with poor survival among patients with colon cancer. Conversely, high MYL9 expression has been associated with poor survival in patients with esophageal squamous cell carcinoma.^11^ Few studies have examined the association between MYL9 expression and cancer. Moreover, no reports regarding the characteristics of MYL9 expression and its clinical significance in PDAC are available. In this study, we investigated the relationship between MYL9 expression and PDAC cell line proliferation.

## 2 METHODS

### 2.1 MYL9 expression in normal and tumor tissues

We analyzed the differences in MYL9 expression levels between normal and tumor tissues using data sets extracted from the Gene Expression database of Normal and Tumor tissues 2 (GENT2) database. GENT2 provides information on gene expression across 72 different tissues. We compared the differences in MYL9 expression between pancreatic cancer and normal tissues.

### 2.2 Collection of cancer tissue samples

The specimens were immediately fixed in 10% formalin for 48 h. For immunohistochemistry against MYL9, 3.5‐μm‐thick sections were prepared from formalin‐fixed, paraffin‐embedded blocks.^18^ A proportion of slides were routinely stained with hematoxylin and eosin (H&E) for pathological evaluation by certified pathologists at our institution. We analyzed samples collected from 101 patients with pancreatic cancer who underwent R0 resection with/without preoperative chemoradiotherapy between April 2007 and September 2013. All study procedures conformed to the provisions of the Declaration of Helsinki (https://www.wma.net/what-we-do/medical-ethics/declaration-of-helsinki/). The use of resected samples was approved by the Human Ethics Review Committee of the Graduate School of Medicine, Osaka University (Osaka, Japan; approval number 664‐7). All patients provided written informed consent for the use of their resected specimens.

### 2.3 Immunohistochemical staining

Immunohistochemical staining was carried out as described previously.^19^ The tissue slides were deparaffinized, hydrated, and incubated overnight at 4°C with the primary antibody: anti‐human MYL9 (1:500; Abcam, Cambridge, MA). Bound antibodies were detected using biotinylated secondary antibodies and diaminobenzidine (Vector Laboratories, Burlingame, CA) as a substrate. The sections were counterstained with hematoxylin.

### 2.4 Cell lines

This study used three human PDAC cell lines (BxPC3, MiaPaCa2, and PSN1). All PDAC cell lines were purchased from the Japan Cancer Research Resources Bank (JCRB; Tokyo, Japan). Each cell line was cultured in Dulbecco's modified Eagle's medium supplemented with 10% fetal bovine serum and 100 units/ml penicillin at 37°C in a humidified incubator with 5% CO2.

### 2.5 Transfection of genes

MYL9‐specific siRNA (Invitrogen) was used to knockdown the MYL9 messenger RNA (mRNA). Transfection of siRNAs into MiaPaCa2, BxPC3, or PSN1 cells was conducted at a final concentration of 20 nM using Lipofectamine RNAiMax (Thermo Fisher Scientific, Tokyo, Japan), using the manufacturer's protocol. The sequence of siRNA used for MYL9 knockdown was as follows: MYL9 siRNA, 5′‐CAGGAGUUUAAGGAGGCUUUCAACA‐3′.

The full‐length human MYL9 cDNA was amplified using PCR and ligated onto the CSII‐CMV‐MCS‐IRES2‐Bsd lentivirus vector (provided by Dr. Miyoshi, RIKEN‐BRC, Japan)^20^ for lentivirus vector construction. The resultant vector was then transfected into 293Ta cells along with the packaging plasmids, using Lipofectamine 3000 (Thermo Fisher Scientific), in accordance with the manufacturer's protocol. The supernatant was collected and filtered for the viral transduction of PSN1 cells with 5 M polybrene (Sigma‐Aldrich, Tokyo, Japan), after 48 h of transfection. Stable pools of cells were obtained by antibiotic selection.

### 2.6 Proliferation assay

A total of 2 × 10^3^ cells per well were seeded in 96‐well plates. Cell proliferation was assessed using a Cell Counting Kit‐8 (Dojindo, Tokyo, Japan), 24, 48, 72, and 96 h after transfection, in accordance with the manufacturer's protocol.

### 2.7 Invasion assay

Invasion assays were performed using 24‐well plates with 8 μm membrane pores and a Matrigel coating (#354480, Corning, NY). We overlaid 1.0 × 10^5^ (MiaPaCa2, BxPC3) or 5.0 × 10^4^ (PSN1) cells onto the Matrigel matrix on a membrane with 8‐mm diameter pores. After 48 h, the invasive cells growing at the bottom of the membrane were fixed with methanol and stained using the Diff‐Quick staining kit (16 920, Sysmex, Hyogo, Japan). Three microscopic fields were randomly selected for cell counting.

### 2.8 Apoptosis assay

To evaluate the level of apoptosis, we performed flow cytometry analysis of Annexin V, along with caspase‐3/7 activation. Annexin V assay was performed using the Annexin V‐FITC Apoptosis Detection Kit (Bio Vision, Milpitas, CA), in accordance with the manufacturer's protocol. After 72 h of each treatment, cells were harvested and stained with Annexin V‐FITC and propidium iodide (Dojindo). Caspase‐3/7 activity was evaluated using the caspase‐Glo® 3/7 Assay Kit from Promega (Madison, WI), and the relative luminescence (RLU) was measured using a GloMax® Microplate Luminometer (Promega). MYL9‐overexpressing PSN1 cells did not induce apoptosis under normal conditions; therefore, 4 nM gemcitabine (Eli Lilly Pharmaceuticals, Indianapolis) was added to the cells. The BD FACS Canto TM II system (BD Biosciences, Tokyo, Japan) was used to perform flow cytometric analysis.

### 2.9 Cell cycle assay

Harvested cells were washed with phosphate buffer saline, fixed with 70% ethanol, treated with RNase, and stained with 25 μg/ml propidium iodide (Dojindo). Flow cytometry was performed using FACSCANTO II (BD Biosciences) after cell staining, and results were analyzed using FlowJo version 10.3 (Tomy Digital Biology, Tokyo, Japan).

### 2.10 Real‐time quantitative reverse transcriptase‐polymerase chain reaction

Total RNA was isolated from the cell lines using TRIzol® RNA Isolation Reagents (Thermo Fisher Scientific), as described previously.^21^ Thereafter, the RNA quality was assessed using a spectrophotometer (RNA concentration > 0.5 μg/μl and OD_260/280_ = 1.8–2.0). The complementary DNA was synthesized using the high‐capacity RNA‐to‐cDNA Kit (Thermo Fisher Scientific), in accordance with the manufacturer's protocol. PCR was performed using the Thunderbird® SYBR® qPCR mix (Toyobo Life Science, Osaka, Japan) in the Light CyclerTM 2.0 System (Roche Applied Science, Tokyo, Japan). Relative expression was evaluated using the comparative CT method. Target gene expression levels were then normalized against the mRNA expression level of glyceraldehyde 3‐phosphate dehydrogenase (GAPDH). Each independent experiment was performed using the independently obtained samples in triplicate to confirm the reproducibility of the data. The sequences of the primers used were as follows:

MYL9, 5′‐ TGACAAGGAGGACCTGCAC‐3′ (forward) and 5′‐ CATCATGCCCTCCAGGTATT‐3′ (reverse); GAPDH, 5′‐ GAAGGTGAAGGTCGGAGT‐3′ (forward) and 5′‐ GAAGATGGTGATGGGATTTC‐3′ (reverse).

### 2.11 Statistical analysis

Clinicopathological parameters were compared using Fisher's exact test, and continuous variables were compared using Student's *t* test. Overall survival (OS) and disease metastasis‐free survival (DMFS) were estimated using the Kaplan–Meier method. Cox's regression was performed for multivariate survival analysis to determine the main independent risk factors for OS or DMFS. Correlations were determined using the Pearson's correlation coefficient. The level of significance was set at *p* ≤ .01 or *p* ≤ .05. All statistical analyses were performed using JMP Statistical Software, version 11 (SAS Institute Inc., Cary, NC).

## 3 RESULTS

### 3.1 Comparison of MYL9 expression in normal and tumor pancreas tissue

**FIGURE 1:**
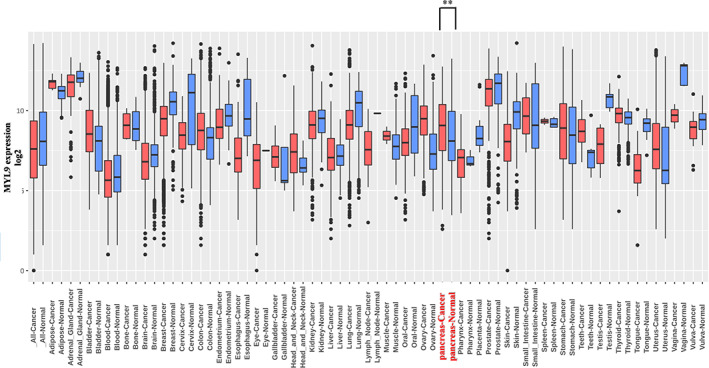
MYL9 expression analysis of various tissue. GENT2 database was used for comparing myosin regulatory light polypeptide 9 (MYL9) expression levels in normal and tumor tissues. In pancreas tissue, MYL9 in pancreatic cancer tissue was upregulated relative to in normal pancreatic tissue (*p* < .01). Each bar represents the cut‐off level for significance (*p < .05, **p < .01)

We studied the expression level of MYL9 using the data sets available in the GENT2 database (Figure 1). GENT2 collects gene expression data from two distinct microarray platforms (Affymetrix U133A and U133Plus2), containing 44 000 and 23 000 samples, respectively. GENT2 evaluates gene expression across 72 different normal and tumor tissues using the results of statistical tests. These data analyses revealed that the expression level of MYL9 is upregulated in pancreatic cancer tissues, compared to that in normal tissues (*p* = .005).

### 3.2 Immunohistochemical staining of MYL9 in patients

To investigate the role of MYL9 in regulating the ability of cancer cells in patients to become malignant, we classified patients with PDAC into three groups, based on MYL9 staining intensities. An immunohistochemistry examination was performed by an observer who was blinded to the clinical data. The observer used a light microscope at a magnification of 100× to grade the MYL9 staining intensity (scale, 0–2). MYL9‐expressing smooth muscle cells of the duodenum (assigned an intensity of 2) were used as a positive control. MYL9 staining was also confirmed in the smooth muscle tissue of blood vessels. Negative cytoplasmic staining was scored as zero (0). Weak cytoplasmic staining compared with the positive control was assigned a score of 1+. A level of staining similar to or stronger than that observed for the positive control was assigned a score of 2+. We identified five fields, including viable cancer ducts. There was no heterogeneity within the tumor area. The MYL9 score was calculated using the sum of the values for five fields. A cut‐off point was set to distinguish tumors with high MYL9 expression levels from those with low expression levels. For the 101 MYL9 specimens, the cut‐off score of four was set as the median score.

**FIGURE 2:**
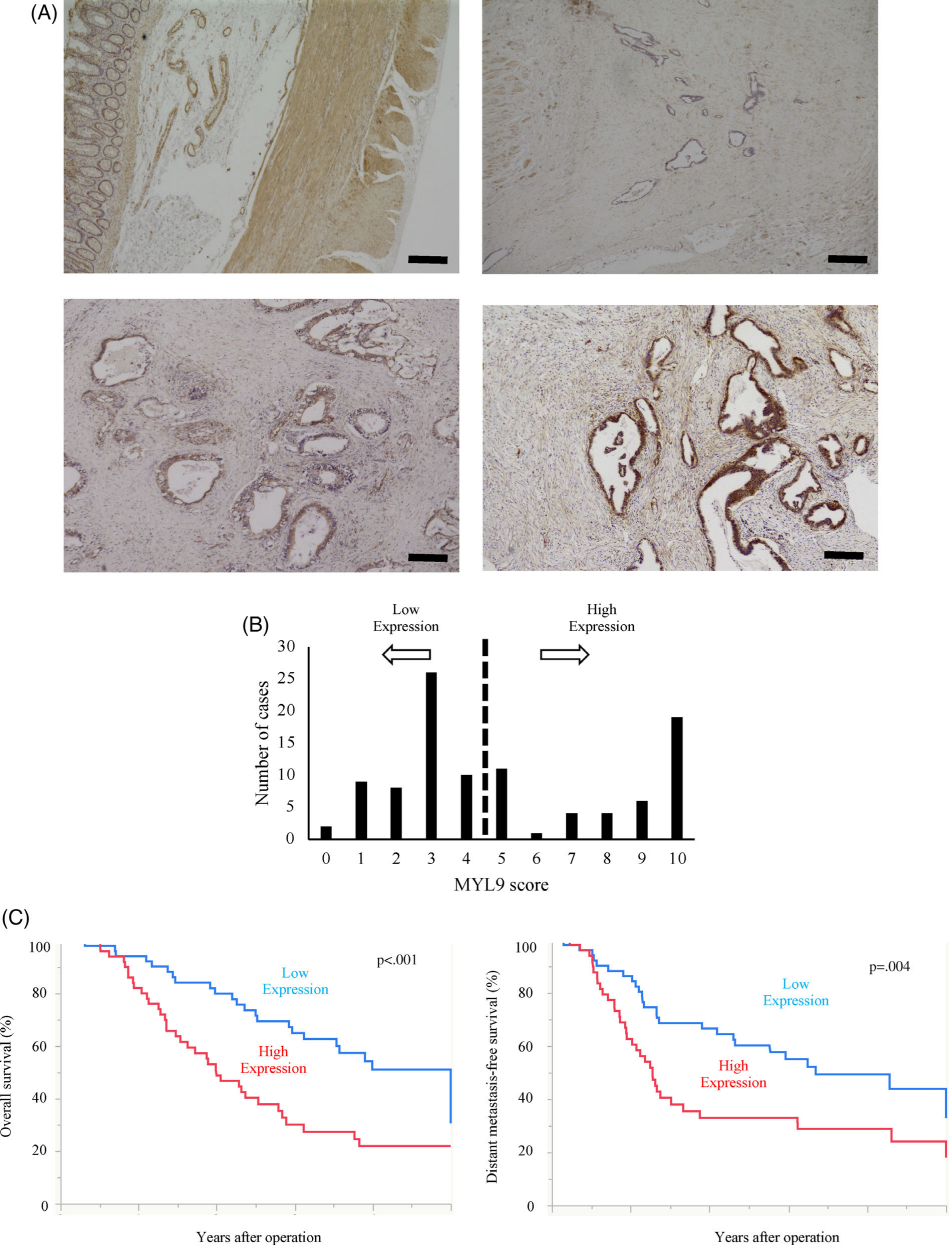
The images are representative of each respective group. (A) Immunohistochemical staining of MYL9 based on intensity. The upper left panel demonstrates the smooth muscle tissue of the duodenum, and is the reference sample, assigned an intensity score of 2. Representative images for each intensity score are shown. For example, we assigned the negative, weakly positive, and strongly positive expression of MYL9 an intensity score of 0 (upper right panel), 1 (bottom left panel), and 2 (bottom right panel), respectively. (B) All patients were divided into MYL9 high and low groups by using the sum of the MYL9 score of five fields. (C) Correlation between MYL9 expression and overall survival and distant‐metastasis‐free survival. Kaplan–Meier curves demonstrated high MYL9 group was positively correlated to poorer prognosis (*p* < .001) and higher relapse rate (*p* = .004) than low MYL9 group

The MYL9 expression score was evaluated based on immunohistochemical analyses of the tumors (Figure 2A). The distribution of the MYL9 expression score is shown in Figure 2B. Tumor factors, such as size, Union for International Cancer Control 8th edition (UICC) stage, and microscopic invasion, were evaluated using the specimens. Univariate analysis of clinicopathological data indicated that patient or tumor factors did not affect MYL9 expression (Table 1). Although there was no correlation between MYL9 expression and tumor factors, MYL9 expression acted as a significant prognostic marker for both DMFS and OS rates in both univariate and multivariate analyses (Tables 2 and 3).

**TABLE 1:** MYL9 expression and clinicopathological features of pancreatic cancer

*n* = 101	MYL9 high (*n* = 50)	MYL9 low (*n* = 51)	*p* value
Age (≥68/<68)	23/27	29/22	.274
Sex (male/female)	30/20	31/20	.938
Tumor size (mm)	30/20	24/27	.155
pT (1,2/3,4)	12/38	15/36	.538
pN (0/1,2)	19/31	15/36	.361
pStage (IA,IB,IIA/IIB,III)	19/31	15/36	.361
v(0/1,2,3)	17/33	16/35	.62
ly(0/1,2,3)	11/39	19/32	.093
ne(0/1,2,3)	42/8	39/12	.342
Preoperative treatment (+/−)	36/14	28/23	.073
Adjuvant chemotherapy (+/−)	29/21	36/15	.186

Abbreviation: UICC, Union for International Cancer Control 8th edition.

**TABLE 2:** The univariate and multivariate analyses of factors associated with overall survival

	Univariate	Multivariate	
*n* = 101	*p* value	Hazard ratio (95% CI)	*p* value
Age (≥68/<68)	.065		
Sex (male/female)	.23		
Tumor size (mm)	**.044**		.774
pT (3,4 /1,2)	.123		
pN (1,2/0)	**.002**		.288
pStage (IIB,III/IA,IB,IIA)	**.002**		NA
v(1,2,3/0)	**.04**	1.80 (1.012–3.173)	**.042**
ly(1,2,3/0)	**.007**		NA
ne(1,2,3/0)	.057		
Preoperative treatment (+/−)	.61		
Adjuvant chemotherapy (+/−)	**<.001**	0.35 (0.207–0.604)	**<.001**
MYL9(high/low)	**.002**	2.03 (1.211–3.446)	**.007**

*Note*: Results with *p*‐values of <.05 were considered statistically significant; boldfaced characters indicate a significant difference.

Abbreviation: UICC, Union for International Cancer Control 8th edition.

**TABLE 3:** The univariate and multivariate analyses of factors associated with distant metastasis free survival

	Univariate	Multivariate	
*n* = 101	*p* value	Hazard ratio (95% CI)	*p* value
Age (≥68/<68)	.253		
Sex (male/female)	.419		
Tumor size (mm)	.156		
pT (3,4 /1,2)	.500		
pN (1,2/0)	**.034**	1.71(1.000–2.898)	**.045**
pStage (IIB,III/IA,IB,IIA)	**.034**		
v(1,2,3/0)	.171		
ly(0/1,2,3)	.392		
ne(0/1,2,3)	.480		
Preoperative treatment (+/−)	.504		
Adjuvant chemotherapy (+/−)	**<.001**	0.37(0.221–0.639)	**<.001**
MYL9(high/low)	**.009**	1.73(1.025–2.949)	**.040**

*Note*: Results with *p*‐values of <.05 were considered statistically significant; boldfaced characters indicate a significant difference.

Abbreviation: UICC, Union for International Cancer Control 8th edition.

Kaplan–Meier curves for DMFS and OS indicated that the MYL9‐positive group demonstrated a significantly poorer prognosis (*p* < .001) and higher relapse rate (*p* = .004) than the negative group (Figure 2C). Multivariate analysis indicated that high expression levels of MYL9 may act as significant prognostic factors for assessing the survival rate (odds ratio [OR]: 2.03; 95% confidence interval [CI]: 1.211–3.446; *p* = .007) (Table 2).

In addition to all patients' analyses, we evaluated the relationship between MYL9 expression and adjuvant chemotherapy in patients who received adjuvant chemotherapy about overall survival and distant metastasis free‐survival as subgroup analysis. Although the MYL9‐positive group tended to have a poorer prognosis (*p* = .088) (Table S1) and higher relapse rate (*p* = .064) (Table S2) than the negative group respectively, there was no significant difference.

### 3.3 MYL9 function is associated with malignant properties of PDAC cells

**FIGURE 3:**
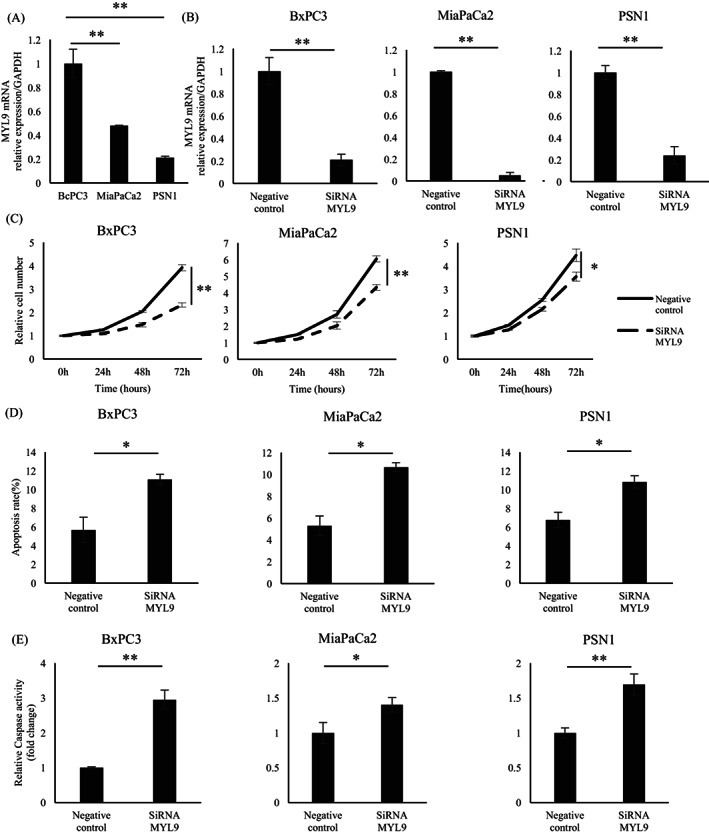
Each bar represents the mean ± *SEM* values of samples measured in triplicate (*p < .05, **p < .01). (A) The mRNA expression levels of MYL9 in BxPC3 cell were higher than MiaPaCa2 and PSN1 cells. (B) The mRNA expression levels of MYL9 in BxPC3, MiaPaCa2, and PSN1 cells: siRNA‐MYL9 led to a significant reduction, compared with the negative control cell. (C) Proliferative activity of BxPC3, MiaPaCa2, and PSN1 cells: siRNA‐MYL9 significantly inhibited cell proliferation, compared with negative control cells. (D) The Annexin V and propidium iodide staining level of MYL9 in BxPC3, MiaPaCa2, and PSN1 cells: siRNA‐MYL9 led to apoptosis, compared with the negative control cell. (E) The caspase‐3/7 activity of MYL9 in BxPC3, MiaPaCa2, and PSN1 cells: siRNA‐MYL9 led to apoptosis, compared to the negative control cell

**FIGURE 4:**
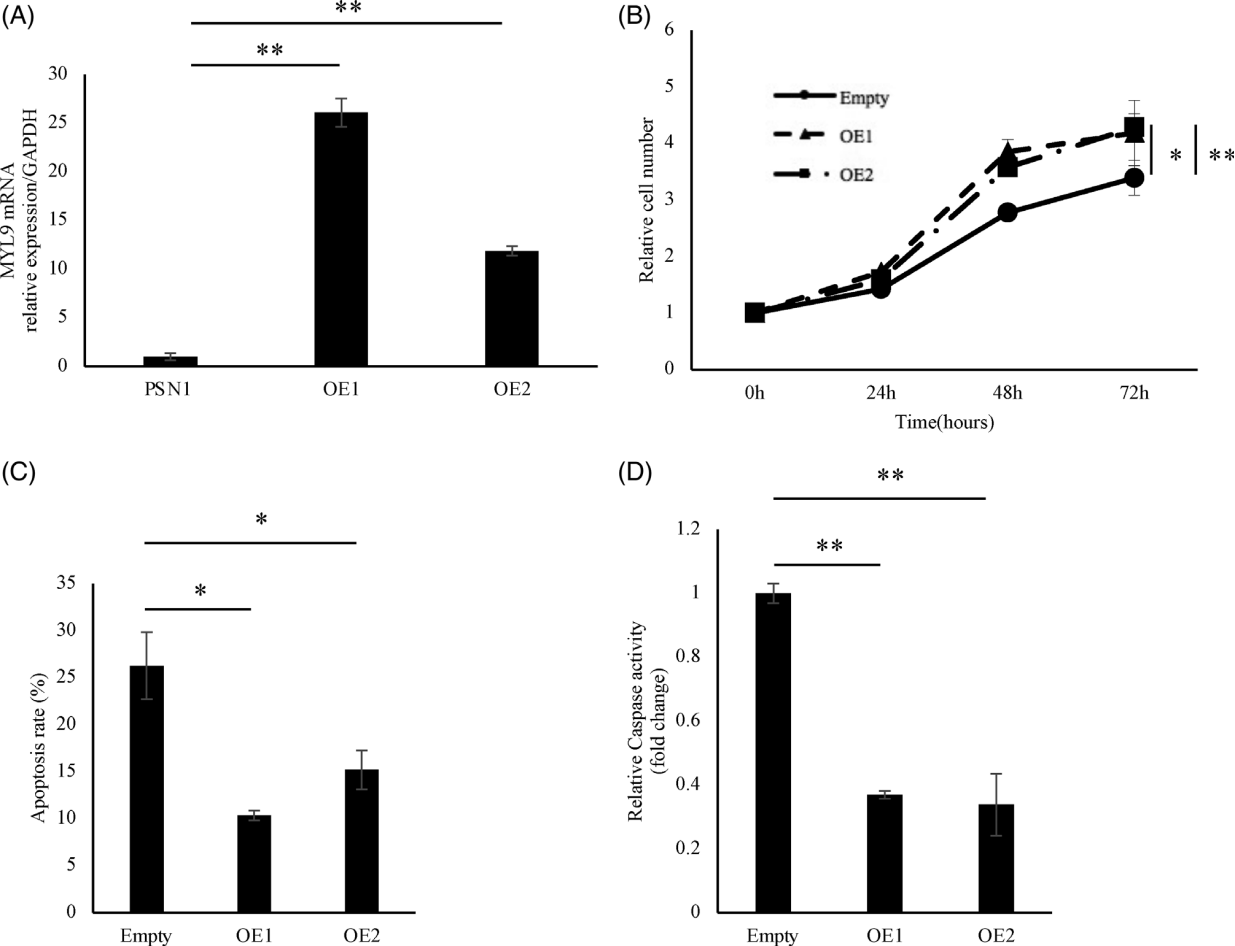
Each bar represents the mean ± *SEM* values of samples measured in triplicate. (*p < .05, **p < .01). Overexpression of myosin regulatory light polypeptide 9 (MYL9) enhanced the malignant potential of pancreatic ductal adenocarcinoma (PDAC) cells. (A) The mRNA expression levels of MYL9 in PSN1 cells: overexpressed‐MYL9 led to significant upregulation, compared with empty cells. (B) Proliferative activity of PSN1 cells: overexpressed‐MYL9 significantly promoted cell proliferation, compared to empty cells. (C) The Annexin V and propidium iodide staining level of PSN1 cells: overexpressed‐MYL9 led to anti‐apoptosis, compared to empty cells. (D) The caspase‐3/7 activity of MYL9 in PSN1 cells: overexpressed‐MYL9 led to anti‐apoptosis, compared with empty cells

To elucidate the involvement of MYL9 in cancer progression, we performed in vitro assays by manipulating *MYL9* gene expression. We evaluated *MYL9* gene expression in PDAC cell lines, including MiaPaCa2, BxPC3, and PSN1 (Figure 3A). The MYL9 expression of MiaPaCa2 and PSN1 cells is 0.47—(*p* = .003) and 0.21—(*p* = .008) fold relative to that of BxPC3 cells, respectively. Next, we performed knockdown experiments using all three PDAC cell lines. RNA interference (RNAi)‐mediated gene silencing suppressed *MYL9* gene expression. The MYL9 expression of siRNA cell in BxPC3, MiaPaCa2, and PSN1 cells was 0.21‐, 0.05‐ and 0.24‐fold relative to that of negative control cell, *p* < .001, *p* < .001 and *p* < .001, respectively (Figure 3B). The knockdown of MYL9 significantly inhibited the cell proliferation of BxPC3, MiaPaCa2, and PSN1 cells (*p* < .001, *p* < .001, and *p* = .02, respectively) (Figure 3C), suggesting that MYL9 directly or indirectly affected the mechanisms involved in PDAC cell apoptosis. Two different apoptosis assays were conducted for all three PDAC cell lines. Here, the knockdown of MYL9 resulted in a remarkably low anti‐apoptotic activity in PDAC cells. The Annexin V assay showed that apoptosis rate in MYL9 siRNA of BxPC3, MiaPaCa2, and PSN1 cells was higher than in negative control siRNA of them (*p* = .01, *p* = .01, and *p* = .02, respectively) (Figure 3D). The caspase3/7 activity of siRNA cell in BxPC3, MiaPaCa2, and PSN1 cells was 2.9‐, 1.4‐, and 1.6‐fold relative to that of negative control cells, (*p* = .005, *p* = .02, and *p* = .002, respectively) (Figure 3E). Cell cycle phase analysis revealed that MYL9 knockdown shortened the lengths of the G0/G1 phase (Figure S1a). The cell invasion assay revealed that the number of cells in the si‐MYL9 transfected group was lower than that in the control group (Figure S2a). Each bar (Figure 3A–E) represents the mean ± *SEM* values of samples measured in triplicate. (*p < .05, **p < .01). Quantitative reverse transcriptase‐polymerase chain reaction (qRT‐PCR) showed that the expression of MYL9 in OE1 and OE2 cells was 26‐ and 11‐fold relative to empty cells (*p* < .01 and *p* < .01, respectively) (Figure 4A). OE1 and OE2 cells had more proliferative activity than empty cells (*p* = .01 and *p* < .01, respectively) (Figure 4B). Additionally, MYL9 overexpression conferred anti‐apoptotic activity to the gemcitabine treatment process. The Annexin V assay showed that apoptosis rate in MYL9 OE1 and OE2 cells was lower than in empty cells (*p* = .02 and *p* = .02, respectively) (Figure 4C). The caspase3/7 activity of MYL9 OE1 and OE2 cells was 0.36‐ and 0.33‐fold relative to that of empty cells, (*p* < .01 and *p* < .01, respectively) (Figure 4D). Cell cycle phase analysis revealed that MYL9 overexpression extended the lengths of the G0/G1 phase (Figure S1b). The cell‐invasion assay revealed that the number of MYL9 overexpressing cells was higher than that of control cells (Figure S2b). Each bar (Figure 4A–D) represents the mean ± *SEM* values of samples measured in triplicate. (*p < .05, **p < .01).

## 4 DISCUSSION

This study resulted in two important findings. First, patients with high MYL9 expression levels exhibited a significantly poorer prognosis than those with low MYL9 expression levels. Second, in PDAC, MYL9 expression levels were associated with the proliferation and invasion abilities of PDAC cells. Previous studies have reported about how MYL9 expression was achieved using proteins from human tissues or total mRNA. Using the data sets extracted from the GENT2 database, we showed that the expression level of MYL9 was upregulated in pancreatic cancer tissues, compared with that in normal tissues. From these findings, we speculate that MYL9 may act as a potential therapeutic target that could prevent the progression of PDAC. Through immunohistochemical analysis, we confirmed that MYL9 was expressed in the tumor‐cell cytoplasm and membrane. We demonstrated that the pattern of MYL9 expression in pancreatic cancer tissues was related to poor prognosis in patients in the overall survival and distant metastasis free survival. Previous studies have demonstrated that the functional role of MYL9 may differ, depending on the cancer type. In our study, we revealed that the expression level of MYL9 was related to cell proliferation. Cell proliferation is compensated by the loss due to apoptosis under normal conditions. Apoptosis is a homeostatic process that helps maintain the cell mass and tissue structure between the processes of proliferation and death.^22^, ^23^ Inhibition of MYL9 expression attenuated cell proliferation; however, the overexpression of MYL9 enhanced cell proliferation. The inhibition of MYL9 induced apoptosis in PDAC cells. Conversely, the overexpression of MYL9 attenuated apoptosis. The overexpression effect between OE1 and OE2 is different. However, the cell proliferation and apoptosis between these two groups are not so different. The function of MYL9 may remain over certain expression levels. These demonstrate that MYL9 is an apoptosis‐related factor, such as Caspase3/7 and Annexin V. In addition, MYL9 plays an important role in maintaining the cytoskeleton; its inhibition may cause instability in the cytoskeleton, thereby inducing apoptosis.

In addition to apoptotic activity, we evaluated the cell cycle upon MYL9 inhibition and overexpression. MYL9 is phosphorylated via ROCK, and MYLK ROCKs‐depleted cells do not undergo division, which indicates that MYL9 blocks the cell cycle. It has been demonstrated that ROCK depletion causes cell‐cycle blockage prior to the G2/M phase. As there is an overall decrease in cells in the S phase, blockage is likely to occur in the G1 phase of the cell cycle. ROCK depletion leads to a significant increase in the number of cells with two nuclei, indicating a failure of cytokinesis.^24^ MYL9 depletion was not associated with cell‐cycle progression in MDA‐MB‐231 cells.^12^ We demonstrated that MYL9 inhibition caused cell cycle arrest in the G0/S phase in pancreatic cancer cells. Conversely, MYL9 overexpression induced the prolongation of the G0/S phase. These results suggest that both apoptosis and cell cycle are associated with cell proliferation via MYL9 in pancreatic cancer cells.

This study has several limitations. First, we evaluated the correlation between total MYL9 protein levels and malignant potential, but not MYL9 phosphorylation. The inhibition of MYL9 phosphorylation has been shown to be beneficial in suppressing invasion in ovarian cancer and hepatoma.^25^, ^26^ Conversely, the dephosphorylation of MYL9 via Y27632 accelerated invasion in noninvasive lung adenocarcinoma cell lines.^27^ The role of MYL9 phosphorylation may differ, based on the type of cancer. Furthermore, the inhibition or overexpression of MYL9 may change the level of MYL9 phosphorylation in pancreatic cancer patients. Second, we demonstrated that the inhibition of MYL9 suppressed the invasive ability of pancreatic cancer cells. However, it is still unclear whether MYL9 regulates cancer‐cell invasion. The invasive ability of cancer cells is related to certain mechanisms, such as cellular adhesion and epithelial‐mesenchymal transition.^28^ We did not investigate the molecular mechanism of cancer‐cell invasion. Additional studies regarding the effect of MYL9 expression on the invasive ability of cancer cells in PDAC are necessary. Third, when knocking down MYL9, this leads to a higher proportion of cells in G2/M phase and would suggest rather an anti‐proliferative function of MYL9. The increasing number of cells in the G2/M phase is due to increasing the number of proliferative or stagnant cells. In this study, we do not evaluate cell cycle markers such as CDK. Knocking down MYL9 may lead to blockage prior to G0/G1 phase and the higher proportion of G2/M phase due to increasing the number of stagnant cells. Since the cause is cell stagnation, the effect of apoptosis has a stronger effect on proliferative capacity than that of cell cycle. Last, we did not evaluate the expression of MYL9 in normal pancreatic tissue. Although normal pancreatic tissue was observed in operative tissue in some cases, other specimens were rich in stromal components, and did not contained normal pancreatic tissue. In this study, we did not evaluate whether the expression of MYL9 in normal pancreas is related to the prognosis of PDAC.

In conclusion, we demonstrated that MYL9 expression in PDAC indicated a greater proliferative ability, which was influenced by apoptosis and the cell cycle. The results of our study suggest that MYL9 expression is a crucial prognostic biomarker and a potential therapeutic target for pancreatic cancer.

## CONFLICT OF INTEREST

Institutional endowments were received in part from Hirotsu Bio Science Inc. (Tokyo, Japan); Kinshu‐kai Medical Corporation (Osaka, Japan); IDEA Consultants, Inc. (Tokyo, Japan); Kyowa‐kai Medical Corporation (Osaka, Japan); and Unitech Co., Ltd. (Chiba, Japan). However, these funders had no role in the procurement of the main experimental equipment, supply expenses, study design, data collection, and analysis, decision to publish, or preparation of this manuscript.

## AUTHOR CONTRIBUTIONS

K.M. had full access to the study data and takes responsibility for the integrity of the data and accuracy of the data analysis. *Conceptualization, Validation, Writing‐Original Drafting*, K.M. *Conceptualization, Formal Analysis, Writing‐Review and Drafting*, S.K. and H.I. *Data Curation*, H.A., M.K., A.A., T.N., Y.I., T.A., and K.G. *Supervision*, M.M., Y.D., and H.E.

## ETHICS STATEMENT

The use of resected samples was approved by the Human Ethics Review Committee of the Graduate School of Medicine, Osaka University (Osaka, Japan; approval number 664‐7). All patients provided written informed consent for the use of their resected specimens.

## Supporting information


**Fig. S1** Each bar represents the mean ± SEM values of samples measured in triplicate. (*p < 0.05, [**p < 0.01]). Cell cycle assay was performed.

**Fig. S2** Each bar represents the mean ± SEM values of samples measured in triplicate. (*p < 0.05, [**p < 0.01]). (a) Invasion ability of PSN1 cells: siRNA‐MYL9 significantly decreased invaded cells, compared to negative control cells. (b) Invasion ability of PSN1 cells: overexpressed‐MYL9 significantly increased invaded cells, compared empty cells.

**Table. S1** The univariate and multivariate analyses with overall survival in patients who received adjuvant chemotherapy

**Table. S2** The univariate and multivariate analyses with distant metastasis free survival in patients who received adjuvant chemotherapy

## Data Availability

There is no available data in this study.
