# Supplementary material for: Clinicopathological significance of MYL9 expression in pancreatic ductal adenocarcinoma
Source: Cancer Rep (Hoboken). 2021 Nov 24;5(10):e1582. doi: 10.1002/cnr2.1582 (PMC9575502; doi:10.1002/cnr2.1582)
Supplement: Supplementary file 1 — Fig. S1 Each bar represents the mean ± SEM values of samples measured in triplicate. (*p < 0.05, [**p < 0.01]). Cell cycle assay was performed. [file CNR2-5-e1582-s001.pdf]

Fig. S1.

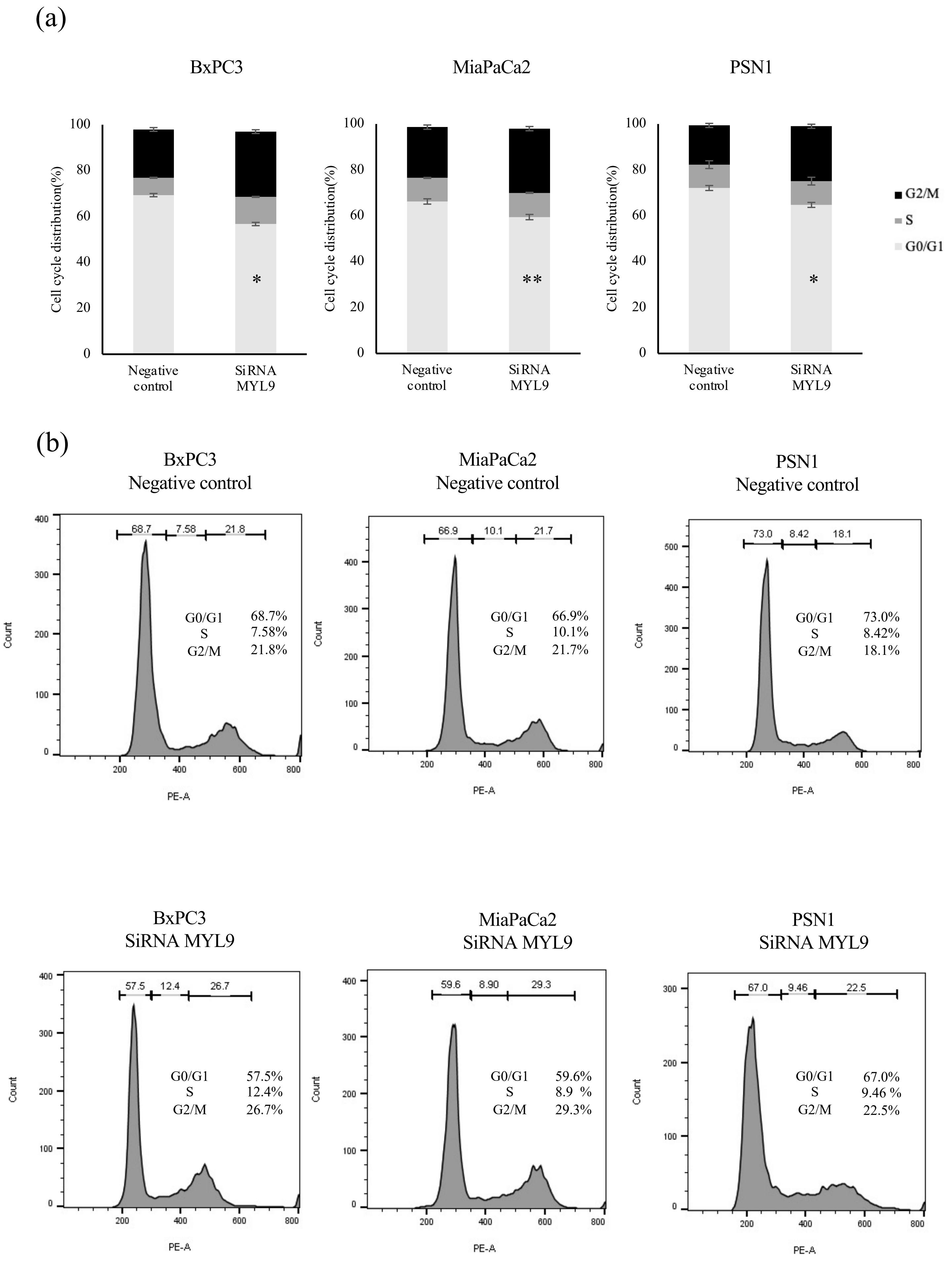

(a)
BxPC3
MiaPaCa2
PSN1
Cell cycle distribution(%)
Negative control
SiRNA MYL9
*
**
*
G2/M
S
G0/G1
(b)
BxPC3
Negative control
G0/G1 68.7%
S 7.58%
G2/M 21.8%
MiaPaCa2
Negative control
G0/G1 66.9%
S 10.1%
G2/M 21.7%
PSN1
Negative control
G0/G1 73.0%
S 8.42%
G2/M 18.1%
BxPC3
SiRNA MYL9
G0/G1 57.5%
S 12.4%
G2/M 26.7%
MiaPaCa2
SiRNA MYL9
G0/G1 59.6%
S 8.9 %
G2/M 29.3%
PSN1
SiRNA MYL9
G0/G1 67.0%
S 9.46 %
G2/M 22.5%
Count
PE-A

(c)

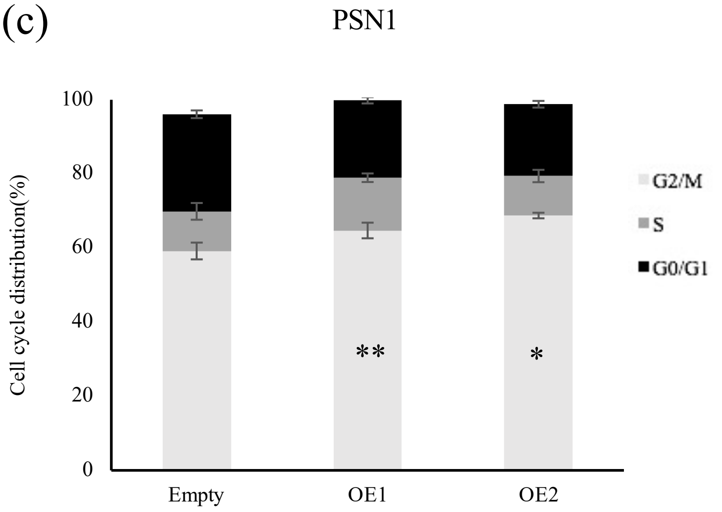

(d)

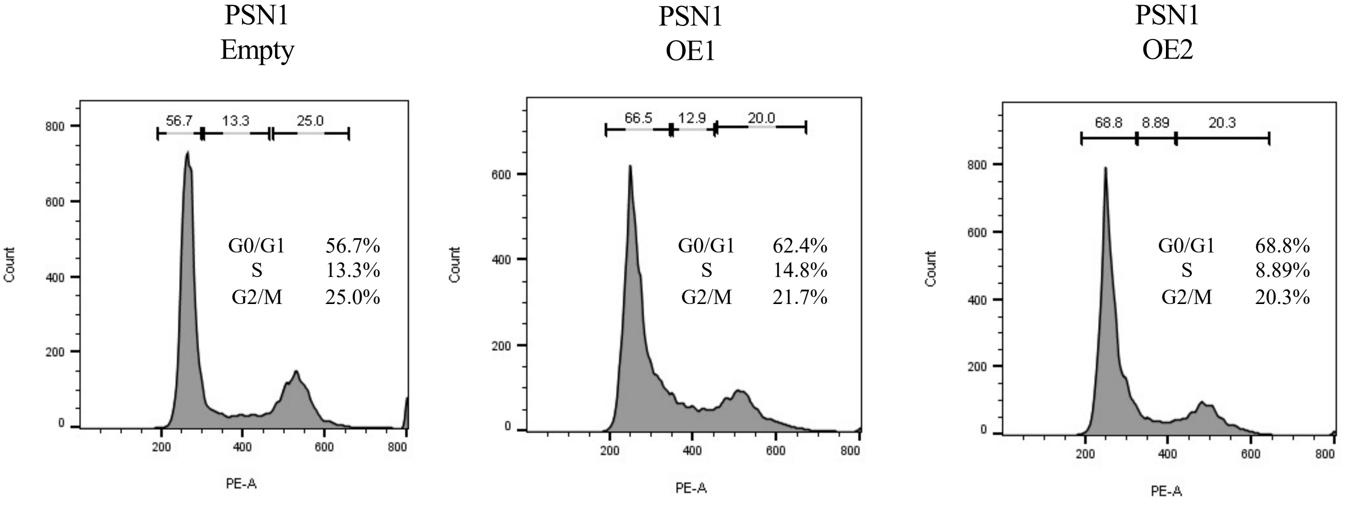

Supplementary figure 1.

(a,b) PDAC cells transfected with negative control or siRNA against MYL9 were stained with propidium iodide and analyzed through flow cytometry. (c,d) Lentivirus-mediated MYL9 overexpressing or negative control cells were stained with propidium iodide and analyzed using flow cytometry. Significant differences of G0/1 phase were observed between negative control and SiMYL9. (*p<0.05, **p<0.01)
