# Supplementary material for: Clinicopathological significance of MYL9 expression in pancreatic ductal adenocarcinoma
Source: Cancer Rep (Hoboken). 2021 Nov 24;5(10):e1582. doi: 10.1002/cnr2.1582 (PMC9575502; doi:10.1002/cnr2.1582)
Supplement: Supplementary file 2 — Fig. S2 Each bar represents the mean ± SEM values of samples measured in triplicate. (*p < 0.05, [**p < 0.01]). (a) Invasion ability of PSN1 cells: siRNA‐MYL9 significantly decreased invaded cells, compared to negative control cells. (b) Invasion ability of PSN1 cells: overexpressed‐MYL9 significantly increased invaded cells, compared empty cells. [file CNR2-5-e1582-s004.pdf]

Fig. S2.

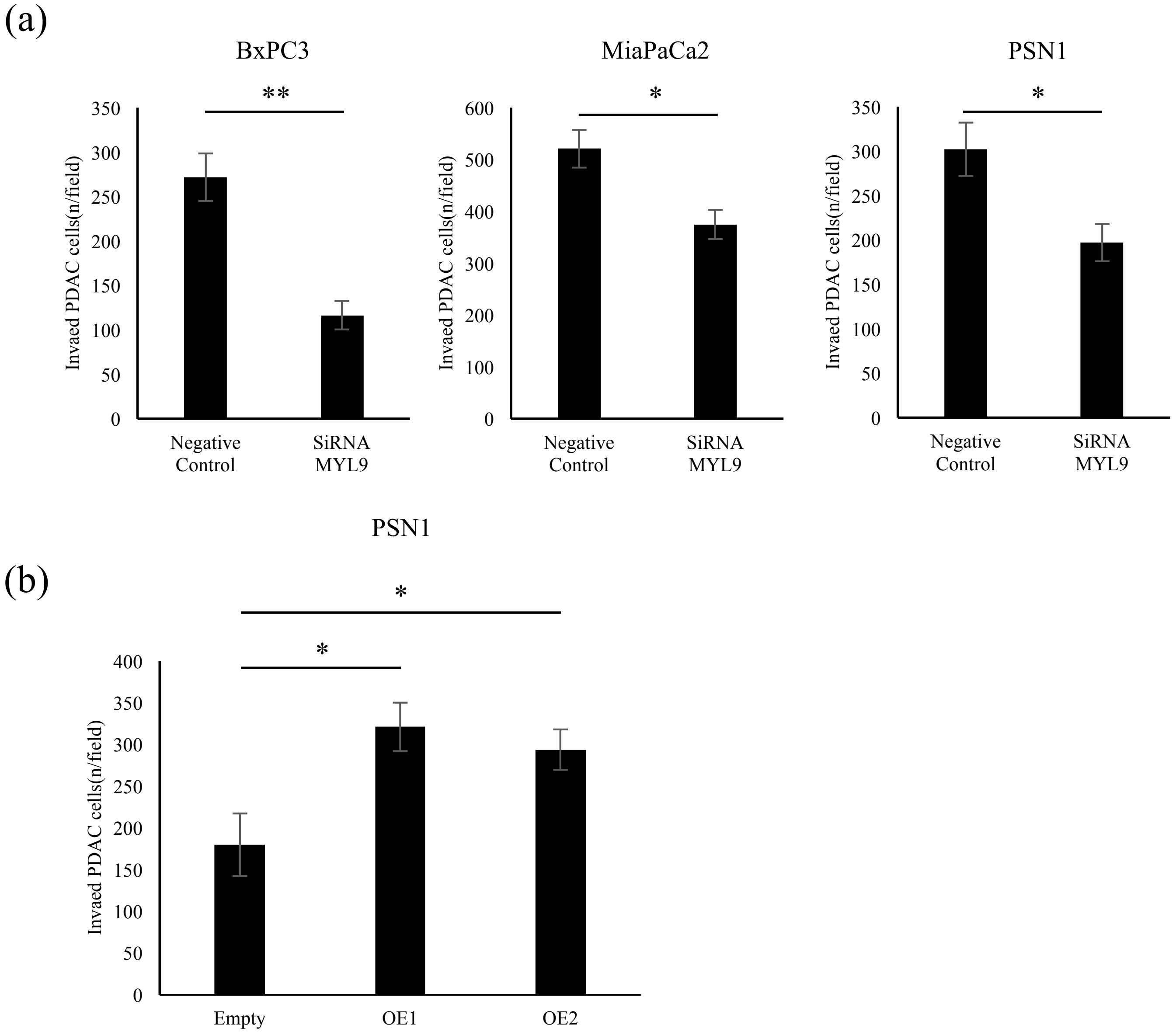

Supplementary figure 2.
(a) The alternation in the number of invasive cells upon MYL9 knockdown. The percentage of change that occurred initially after the treatment is shown. Invasiveness in PDAC cells decreased upon transfection with siRNA against MYL9. (b) The alteration in the number of invasive cells expressing lentivirus-mediated MYL9. The percentage of change that occurred initially after treatment is shown. Invasiveness in PDAC cells increased with lentivirus-mediated overexpression of MYL9. Each bar represents the mean ± SEM of samples measured in triplicate (*$p<0.05$, **$p<0.01$).
