# Supplementary material for: Clinicopathological significance of MYL9 expression in pancreatic ductal adenocarcinoma
Source: Cancer Rep (Hoboken). 2021 Nov 24;5(10):e1582. doi: 10.1002/cnr2.1582 (PMC9575502; doi:10.1002/cnr2.1582)
Supplement: Supplementary file 4 — Table. S2 The univariate and multivariate analyses with distant metastasis free survival in patients who received adjuvant chemotherapy [file CNR2-5-e1582-s002.docx]

**Table S2.** The univariate and multivariate analyses of factors associated with distant metastasis free survival in patients who received adjuvant chemotherapy

|  | Univariate | | Multivariate | | | |
| --- | --- | --- | --- | --- | --- | --- |
| *n* = 65 | *p* value | |  | hazard ratio (95% CI) | *p* value | |
| Age (≥68/<68) | 0.797 |  |  | |  |  |
| Sex (male/female) | 0.747 |  |  | |  |  |
| Tumor size (mm) | 0.525 |  |  | |  |  |
| pT (3,4 /1,2) | 0.598 |  |  | |  |  |
| pN (1,2/0) | 0.396 |  |  | |  |  |
| pStage (IIB,III/IA,IB,IIA) | 0.396 |  |  | |  |  |
| v(1,2,3/0) | 0.778 |  |  | |  |  |
| ly(1,2,3/0) | 0.513 |  |  | |  |  |
| ne(1,2,3/0) | 0.452 |  |  | |  |  |
| Preoperative treatment (+/-) | **0.048** |  |  | |  |  |
| MYL9(high/low) | 0.064 |  |  | |  |  |

UICC, Union for International Cancer Control 8^th^ edition

Results with *p*-values of < 0.05 were considered statistically significant; boldfaced characters indicate a significant difference
